# Comprehensive analysis of untargeted metabolomics and lipidomics in girls with central precocious puberty

**DOI:** 10.3389/fped.2023.1157272

**Published:** 2023-05-12

**Authors:** Hong-Ying Zhao, Ya-Rong Zhang, Ran Zhang, Yu-Ting Li, Rong-Lian Guo, Wen-Sheng Shi

**Affiliations:** ^1^ Pediatric Department, Zhongshan Hospital, Xiamen University, Xiamen, China; ^2^ School of Medicine, Xiamen University, Xiamen, China; ^3^ Center of Clinical Laboratory, Zhongshan Hospital, Xiamen University, Xiamen, China

**Keywords:** central precocious puberty, metabolomics, lipidomics, metabolites, diagnostic value

## Abstract

**Objective:**

Central precocious puberty (CPP) is a rare condition that causes early sexual development in children. Although the cure is effective, the etiology of central precocious puberty is unclear.

**Methods:**

In total, 10 girls with central precocious puberty and same number of age-matched female controls were enrolled. Plasma samples were collected from each participant and subjected to untargeted metabolomics and lipidomics. Student's *t*-tests were employed to compare the mean of each metabolite and lipid. Furthermore, orthogonal partial least-squares discriminant analysis was conducted and the variable importance in the projection was calculated to identify differentially expressed metabolites or lipids. Subsequent bioinformatics was conducted to investigate the potential function of differentially expressed metabolites and lipids.

**Results:**

Fifty-nine differentially expressed metabolites were identified based on the criteria used (variable importance in the projection >1 and a *P* value < 0.05). Kyoto Encyclopedia Genes and Genome (KEGG) enrichment analysis showed that differentially expressed metabolites were enriched in four pathways: beta-alanine metabolism, histidine metabolism, bile secretion, and steroid hormone biosynthesis. As for the lipidomics, 41 differentially expressed lipids were observed and chain length analysis and lipid saturation analysis yielded similar results. Significant differences between the two groups were only observed in (O-acyl) ω-hydroxy fatty acids (OAHFA).

**Conclusion:**

The present study showed that antibiotic overuse, increased meat consumption, and obesity may have potential roles in the development of central precocious puberty in girls. Several metabolites have diagnostic value but further research is required.

## Introduction

Central precocious puberty (CPP) refers to a rare condition that causes early sexual development in children. Normally, children are considered to have precocious puberty if it begins before 8 years of age in girls and before 9 years of age in boys. Sexual development, growth, and even behavioral changes can be attributed to activation of the hypothalamic–pituitary–gonadal axis, which is a complex central nervous system network (1, 2). The central nervous system network includes several neuronal populations in the hypothalamus, the pituitary gonadotrophic cells, and the gonads. The key molecular messenger of puberty is hypothalamic decapeptide gonadotropin-releasing hormone (GnRH), and the release of GnRH causes the synthesis and release of two gonadotrophins, luteinizing hormone and follicle stimulating hormone, into the peripheral circulation. The gonadotrophins in the peripheral circulation stimulate the gonads to produce sex steroid hormones and gametes, which leads to pubertal changes, such as the growth of pubic and underarm hair, height growth, and the development of breasts in girls.

There are existing studies reporting the epidemiological characteristics of CPP, and they showed that the incidence of CPP varies greatly between countries and by gender. Kim et al. (3) conducted a study based on data from a national registry in South Korea and reported an overall incidence of CPP of 12.28 per 10,000 persons (26.28 in girls and 0.7 in boys). In a study in a French population, the incidence of CPP was 2.68 per 10,000 in girls and 0.24 per 10,000 in boys, according to a national insurance database (4). By contrast, the incidence of CPP was relatively low in a Spanish population (0.217 in girls and 0.023 in boys) in a study involving 34 pediatric endocrinology units throughout the country (5). A prospective study in Denmark from 1998 to 2017 revealed a steady growth in the incidence of CPP during the observation period, indicating that CPP is a public health matter that requires immediate intervention to preserve the well-being of children (6).

CPP is treatable with several medications that block the production of luteinizing hormone and follicle stimulating hormone, and consequently stop the progression of CPP. The safest and most effective option is the use of long-acting GnRH analogs, and an extended-release formulation of GnRH analogs is now available, which provides a more convenient solution for both patients and healthcare providers (7, 8). Although treatment for CPP is effective and has been used worldwide, the etiology of CPP is a complex interaction between genetic factors, environmental exposure, nutritional status (9–11), and other potential influencers. Metabolic status is closely associated with puberty (12). As an example, the average age of menarche for girls in the early 19th century was 17 years but had dramatically declined to 13 years by the middle of the 20th century. The change in the age of menarche can be largely attributed to improved nutrition and better socioeconomic conditions (13).

Several metabolites were altered in urine samples of CPP patients; also, the catecholamine metabolic pathway, tryptophan metabolic pathway, and tricarboxylic acid (TCA) cycle were also altered (14). Recently, Li et al. (15) conducted an integrated analysis of proteomics and metabolomics in girls with CPP. The results showed that differentially expressed metabolites (DEM) were enriched in lipid and taurine pathways, while several proteins (including MMP9, TIMP1, and SPP1) were associated with puberty development. Taken together, previous research findings indicate that lipid metabolism and nutritional status, especially obesity, have an important role in the development of CPP.

To investigate changes in lipids and metabolites in CPP patients, we conducted a comprehensive investigation combining untargeted metabolomics and lipidomics. We also explored pathways and potential biomarkers for the etiology and diagnosis of CPP.

## Materials and methods

### Study participants

Enrollment of study participants was conducted between October 2021 and August 2022 in the Pediatric Department of Zhongshan Hospital Xiamen University. The inclusion of female CPP patients was based on a consensus report on the diagnosis and treatment of CPP published by the Chinese Medical Association (16). Study participants who met the following criteria were included in the CPP group: (1) breast development before 8 years of age; (2) linear growth acceleration involving a higher than normal annual growth rate; (3) progressive bone age more than 1 year above the chronological age; (4) pelvic ultrasound of the uterus revealing a length of 3.4 cm–4.0 cm, an ovarian volume of 1–3 ml, and the presence of multiple follicles ≥4 mm in diameter; and (5) an luteinizing hormone peak ≥5 IU/l and an luteinizing hormone/follicle stimulating hormone peak ≥0.6 after GnRH stimulation.

Healthy controls were selected from female children who visited the hospital for routine medical examination, matched by age to CPP patients. In total, 10 female CPP patients and the same number of female controls were included in the present study. The study was approved by the Ethics Committee of Zhongshan Hospital, Xiamen University. Informed consent was obtained from the legal guardians of each study participant. This study was carried out in accordance with the Declaration of Helsinki.

### Sample collection

Heparin-anticoagulated blood samples were collected from each study participant in the morning after fasting for at least 8 h. After collection, samples were centrifuged at 4 °C and 3,000 rpm for 10 min to separate plasma. Plasma was immediately transferred into an Eppendorf tube and stored at −80 °C before subsequent analyses.

### Metabolomics

The plasma samples were thawed at 4 °C and 100 µl aliquots were mixed with 400 µl of cold methanol/acetonitrile (1:1, v/v) to remove the protein. The mixture was centrifuged for 20 min (14,000*g*, 4 °C). The supernatant was dried in a vacuum centrifuge (17). For LC-MS analysis, the samples were re-dissolved in 100 µl acetonitrile/water (1:1, v/v) solvent and centrifuged at 14,000*g* at 4 °C for 15 min, then the supernatant was injected. Untargeted metabolomics analyses were performed using an ultra-high performance liquid chromatography (UHPLC) (1290 Infinity LC, Agilent Technologies) coupled to a quadrupole time-of-flight (AB Sciex TripleTOF 6600).

For HILIC separation, samples were analyzed using a 2.1 mm × 100 mm ACQUIY UPLC BEH Amide 1.7 µm column (waters, Ireland). In both ESI positive and negative modes, the mobile phase contained *A* = 25 mM ammonium acetate and 25 mM ammonium hydroxide in water and *B* = acetonitrile. The gradient was 95% B for 0.5 min and was linearly reduced to 65% in 6.5 min, and then was reduced to 40% in 1 min and kept for 1 min, and then increased to 95% in 0.1 min, with a 3 min re-equilibration period employed. The ESI source conditions were set as follows: Ion Source Gas1 (Gas1) as 60, Ion Source Gas2 (Gas2) as 60, curtain gas (CUR) as 30, source temperature: 600 °C, IonSpray Voltage Floating (ISVF) ± 5,500 V. In MS only acquisition, the instrument was set to acquire over the *m*/*z* range 60–1,000 Da, and the accumulation time for TOF MS scan was set at 0.20 s/spectra. In auto MS/MS acquisition, the instrument was set to acquire over the *m*/*z* range 25–1,000 Da, and the accumulation time for product ion scan was set at 0.05 s/spectra. The product ion scan is acquired using information dependent acquisition (IDA) with high sensitivity mode selected. The parameters were set as follows: the collision energy (CE) was fixed at 35 V with ±15 eV; declustering potential (DP), 60 V (+) and −60 V (−); exclude isotopes within 4 Da, candidate ions to monitor per cycle: 10.

Raw MS data were converted into MzXML files using ProteoWizard MSConvert before being imported into freely available XCMS software. The following parameters were used to identify peaks: centWave *m*/*z* = 0 ppm, peakwidth = *c* (10, 60), and prefilter = *c* (10, 100). For peak grouping, the following parameters were used: bw = 5, mzwid = 0.025, and minfrac = 0.5. CAMERA (Collection of Algorithms of MEtabolite pRofile Annotation) was used for annotation of isotopes and adducts. In the extracted ion features, only variables having more than 50% of the nonzero measurement values in at least one group were kept. Compound identification of metabolites was performed by comparing the accuracy of *m*/*z* values (<10 ppm), and by analyzing MS/MS spectra using an in-house database established with available authentic standards. After sum-normalization, the processed data were analyzed using R package (ropls), and multivariate data analysis was carried out, including Pareto-scaled principal component analysis and orthogonal partial least-squares discriminant analysis (OPLS-DA). Seven-fold cross-validation and response permutation testing was used to evaluate the robustness of the model. The variable importance in the projection value of each variable in the OPLS-DA model was calculated to indicate its contribution to the classification. The Student's t*-*test was applied to determine the significance of differences between two groups of independent samples. Variable importance in the projection (VIP) > 1 and a *P* value < 0.05 were used to screen significantly changed metabolites. Pearson's correlation analysis was performed to determine the correlation between two variables.

### Lipidomics

Lipids were extracted according to Methyl-tert-Butyl Ether (MTBE)method (18). Briefly, a 200-µl volume of water was added to 30 mg sample and vortexed for 5 s. Subsequently, 240 µl of precooling methanol was added and the mixture vortexed for 30 s. After that, 800 µl of MTBE was added and the mixture was ultrasound 20 min at 4 °C followed by sitting still for 30 min at room temperature. The solution was centrifuged at 14,000*g* for 15 min at 10 °C and the upper organic solvent layer was obtained and dried under nitrogen. Reverse phase chromatography was selected for LC separation using a CSH C18 column (1.7 µm, 2.1 mm × 100 mm, Waters). The lipid extracts were re-dissolved in 200 µl 90% isopropanol/acetonitrile, centrifuged at 14,000×*g* for 15 min and 3 µl of the sample was injected. Solvent A was acetonitrile–water (6:4, v/v) with 0.1% formic acid and 0.1 Mm ammonium formate, and solvent B was acetonitrile–isopropanol (1:9, v/v) with 0.1% formic acid and 0.1 Mm ammonium formate. The initial mobile phase was 30% solvent B at a flow rate of 300 µl/min. It was held for 2 min and then linearly increased to 100% solvent B in 23 min, followed by equilibrating with 5% solvent B for 10 min.

Mass spectra were acquired by Q-Exactive Plus in both positive and negative modes. ESI parameters were optimized and preset for all measurements as follows: a source temperature of 300 °C; a capillary temperature of 350 °C; an ion spray voltage of 3,000 V; and an S-lens RF level of 50%. The scan range of the instruments was set at 200–1,800 *m*/*z*. “Lipid Search” is a search engine for the identification of lipid species based on MS/MS math. LipidSearch contains more than 30 lipid classes and more than 1,500,000 fragment ions in the database. Both mass tolerance for precursor and fragment were set to 5 ppm (19).

## Results

### Metabolomics

**Figure 1:**
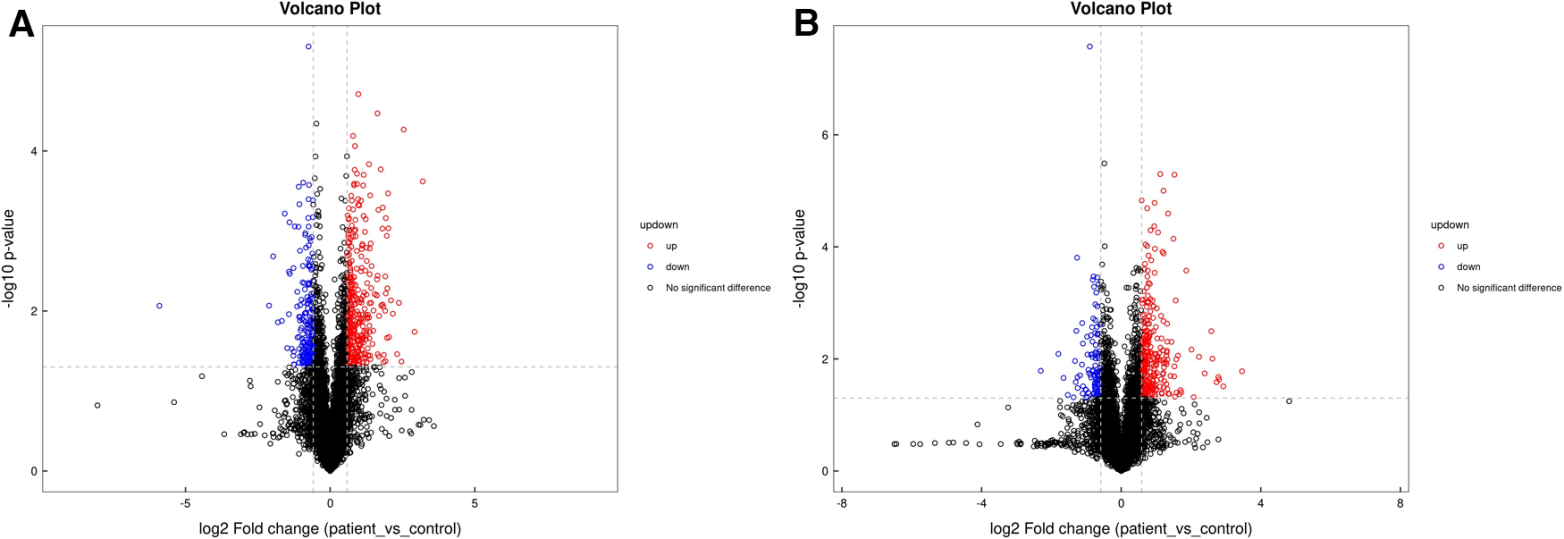
Volcano plot of metabolites in (**A**) positive ion mode and (**B**) negative ion mode.

**Figure 2:**
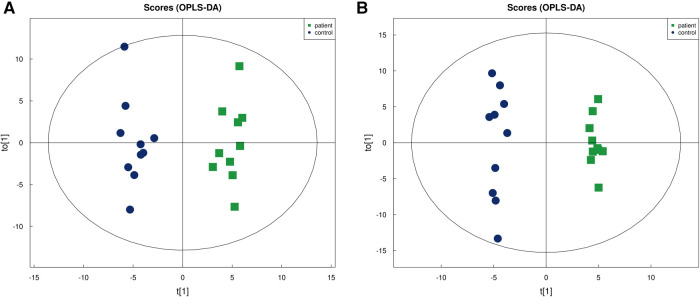
OPLS-DA diagram of metabolites in (**A**) positive ion mode and (**B**) negative ion mode.

Untargeted metabolomics analysis identified 866 metabolites in positive ion mode and 302 in negative ion mode. Based on the results of Student's t-tests and fold change analysis (FC), metabolites were selected with an FC > 1.5 or <0.67 and a *P* value <0.05 (Figures 1A,B). In OPLS-DA, R^2^Y and Q^2^ were 0.962 and 0.574, respectively, in positive ion mode, and the corresponding parameters in negative ion mode were 0.991 and 0.496, respectively. As can be seen in Figures 2A,B, the CPP of patients and controls were clearly separated in both positive and negative ion modes, indicating that OPLS-DA was effective. A permutation test was conducted to evaluate the robustness of OPLS-DA, showing that R^2^Y and Q^2^ gradually decreased in both positive and negative ion modes, and were lower than those of the actual dataset, suggesting that there was no overfitting of the model.

**Figure 3:**
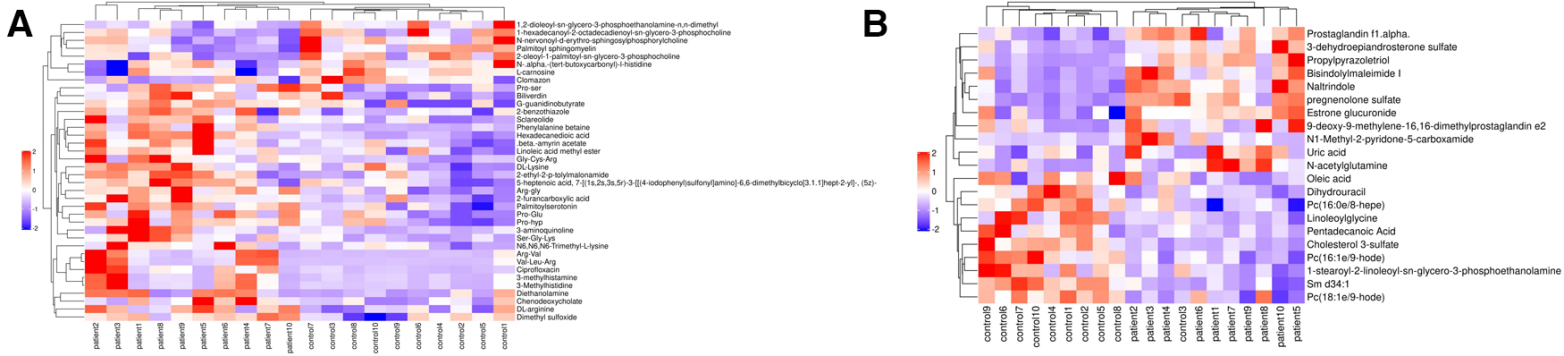
Heatmap of DEMs in (**A**) positive ion mode and (**B**) negative ion mode.

Furthermore, we applied the following criteria to identify DEM if they had a variable importance in the projection calculated from OPLS-DA > 1 and a *P* value <0.05. In total, 38 metabolites in the positive ion mode and 21 in the negative ion mode were identified as DEMs, and detailed information of DEMs is displayed in Table 1. Hierarchical clustering based on DEMs showed clear separation between groups and similarity inside groups in both positive negative ion modes (Figures 3A,B). Kyoto Encyclopedia Genes and Genome (KEGG) enrichment analysis was also performed and the results showed that DEMs were enriched in four pathways: beta-alanine metabolism, histidine metabolism, bile secretion, and steroid hormone biosynthesis.

**Table 1:** List of differentially expressed metabolites in CPP patients and controls.

Name	VIP	Fold change	*p*-value	*m*/*z*	rt(s)
Sm d34:1	1.555	0.703	<0.001	761.584	181.877
Pc(16:1e/9-hode)	6.482	0.732	<0.001	816.577	153.244
1-Stearoyl-2-linoleoyl-sn-glycero-3-phosphoethanolamine	2.872	0.753	0.001	742.541	153.496
Cholesterol 3-sulfate	1.276	0.591	0.001	465.305	48.634
Naltrindole	6.184	2.341	0.001	413.201	44.949
Propylpyrazoletriol	5.345	4.032	0.001	385.170	55.164
Pregnenolone sulfate	1.264	2.397	0.002	395.190	40.971
Pc(16:0e/8-hepe)	1.855	0.820	0.003	840.578	149.398
Linoleoylglycine	1.035	0.617	0.003	673.531	182.497
Bisindolylmaleimide I	1.205	2.090	0.003	411.185	39.021
Prostaglandin f1.alpha.	1.277	1.439	0.004	337.239	173.830
Estrone glucuronide	1.102	1.610	0.004	427.180	205.852
Pc(18:1e/9-hode)	3.618	0.745	0.006	844.605	151.494
3-Dehydroepiandrosterone sulfate	5.198	3.504	0.008	367.159	41.830
Dihydrouracil	1.071	0.658	0.009	113.024	109.900
Pentadecanoic Acid	1.693	0.545	0.011	241.218	41.372
N-Acetylglutamine	2.237	2.580	0.018	187.073	395.001
Uric acid	1.539	1.623	0.020	167.021	326.624
Oleic acid	1.362	0.641	0.040	281.249	110.329
N1-Methyl-2-pyridone-5-carboxamide	1.339	3.605	0.044	168.078	371.573
9-Deoxy-9-methylene-16,16-dimethylprostaglandin e2	1.868	1.722	0.046	377.270	184.473
Arg-gly	1.745	1.719	<0.001	232.139	419.574
Hexadecanedioic acid	2.379	1.768	<0.001	251.198	159.835
Palmitoylserotonin	1.524	1.518	0.001	415.353	158.563
Phenylalanine betaine	1.677	5.992	0.003	208.132	194.514
Pro-hyp	13.525	1.495	0.003	229.118	416.029
2-Furancarboxylic acid	1.009	1.530	0.004	112.999	416.712
Palmitoyl sphingomyelin	22.173	0.713	0.005	703.572	178.891
2-Ethyl-2-p-tolylmalonamide	1.092	1.334	0.007	221.152	182.014
DL-arginine	16.452	1.389	0.007	175.119	485.522
2-Oleoyl-1-palmitoyl-sn-glycero-3-phosphocholine	8.001	0.779	0.008	782.566	35.006
G-guanidinobutyrate	8.182	2.067	0.009	146.091	377.085
Gly-Cys-Arg	1.152	2.501	0.010	335.152	417.720
Pro-Glu	1.373	1.512	0.010	286.138	411.133
Beta-amyrin acetate	1.446	1.427	0.012	491.366	156.941
Ser-Gly-Lys	1.443	2.467	0.012	291.152	457.613
Sclareolide	1.044	1.453	0.013	501.388	34.378
Pro-ser	1.618	1.682	0.013	203.112	364.986
2-Benzothiazole	1.088	1.434	0.014	136.020	75.490
Linoleic acid methyl ester	2.851	1.656	0.016	263.235	113.249
Ciprofloxacin	2.532	5.251	0.018	332.143	385.032
3-Aminoquinoline	2.889	1.907	0.020	145.075	77.301
L-carnosine	5.313	0.815	0.020	156.076	367.755
Diethanolamine	2.287	1.785	0.021	88.075	193.834
N-Nervonoyl-d-erythro-sphingosylphosphorylcholine	2.722	0.731	0.022	813.679	197.183
Dimethyl sulfoxide	7.515	1.122	0.023	79.021	54.894
Arg-Val	1.322	6.989	0.023	238.163	255.750
1,2-Dioleoyl-sn-glycero-3-phosphoethanolamine-n,n-dimethyl	2.420	0.682	0.026	772.580	148.172
Val-Leu-Arg	1.391	6.652	0.026	194.137	258.339
5-heptenoic acid, 7-[(1s,2s,3s,5r)-3-[[(4-Iodophenyl)sulfonyl]amino]- 6,6-dimethylbicyclo[3.1.1]hept-2-yl]-, (5z)-	1.101	1.390	0.029	249.183	202.181
Chenodeoxycholate	1.664	1.673	0.031	357.275	161.802
N-.alpha.-(tert-butoxycarbonyl)-l-histidine	4.263	0.803	0.039	110.071	368.178
3-Methylhistidine	10.214	3.294	0.039	170.092	370.417
Biliverdin	2.544	1.614	0.042	583.249	136.898
3-methylhistamine	1.645	3.177	0.043	126.101	370.388
N6,N6,N6-Trimethyl-L-lysine	2.697	1.677	0.045	189.158	496.843
1-Hexadecanoyl-2-octadecadienoyl-sn-glycero-3-phosphocholine	19.831	0.838	0.047	758.567	148.736
Clomazon	1.418	0.698	0.049	240.101	213.994
DL-Lysine	1.810	1.171	0.050	147.112	495.908

### Lipidomics

**Figure 4:**
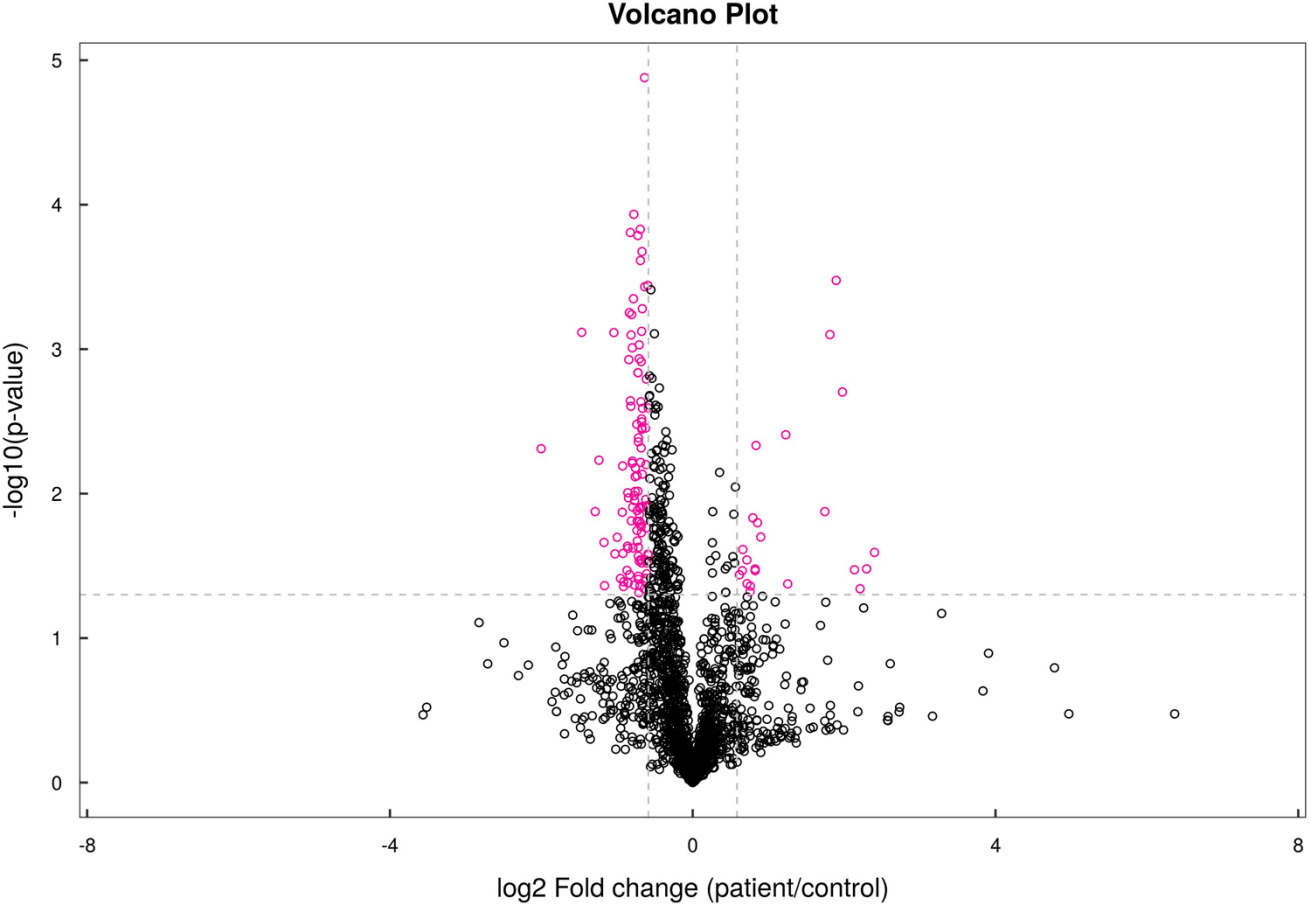
Volcano plot of lipids.

**Figure 5:**
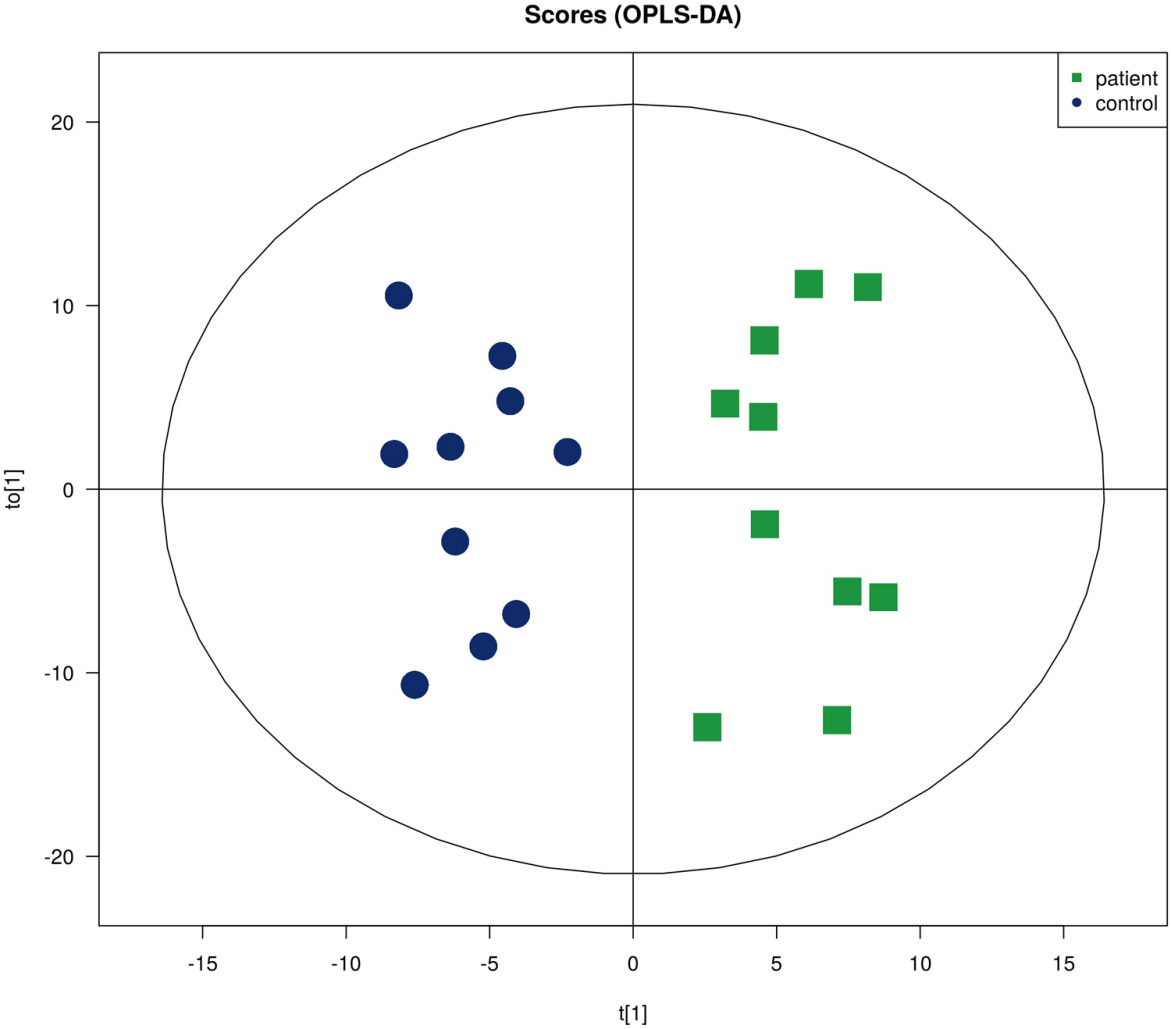
OPLS-DA diagram of lipids.

In total, lipidomics analysis identified 1,831 lipid species in 41 lipid classes. Similarly, FC analysis and Student's *t*-tests were used to find lipid species that were significantly different between CPP patients and controls. The pink dots in the volcano plot show lipid species with an FC > 1.5 or <0.67 and a *P* value < 0.05 (Figure 4). OPLS-DA yielded R^2^Y and Q^2^ values of 0.896 and 0.420, respectively, suggesting that the model was stable and effective. In addition, Figure 5 of OPLS-DA shows a clear separation between CPP patients and controls.

**Figure 6:**
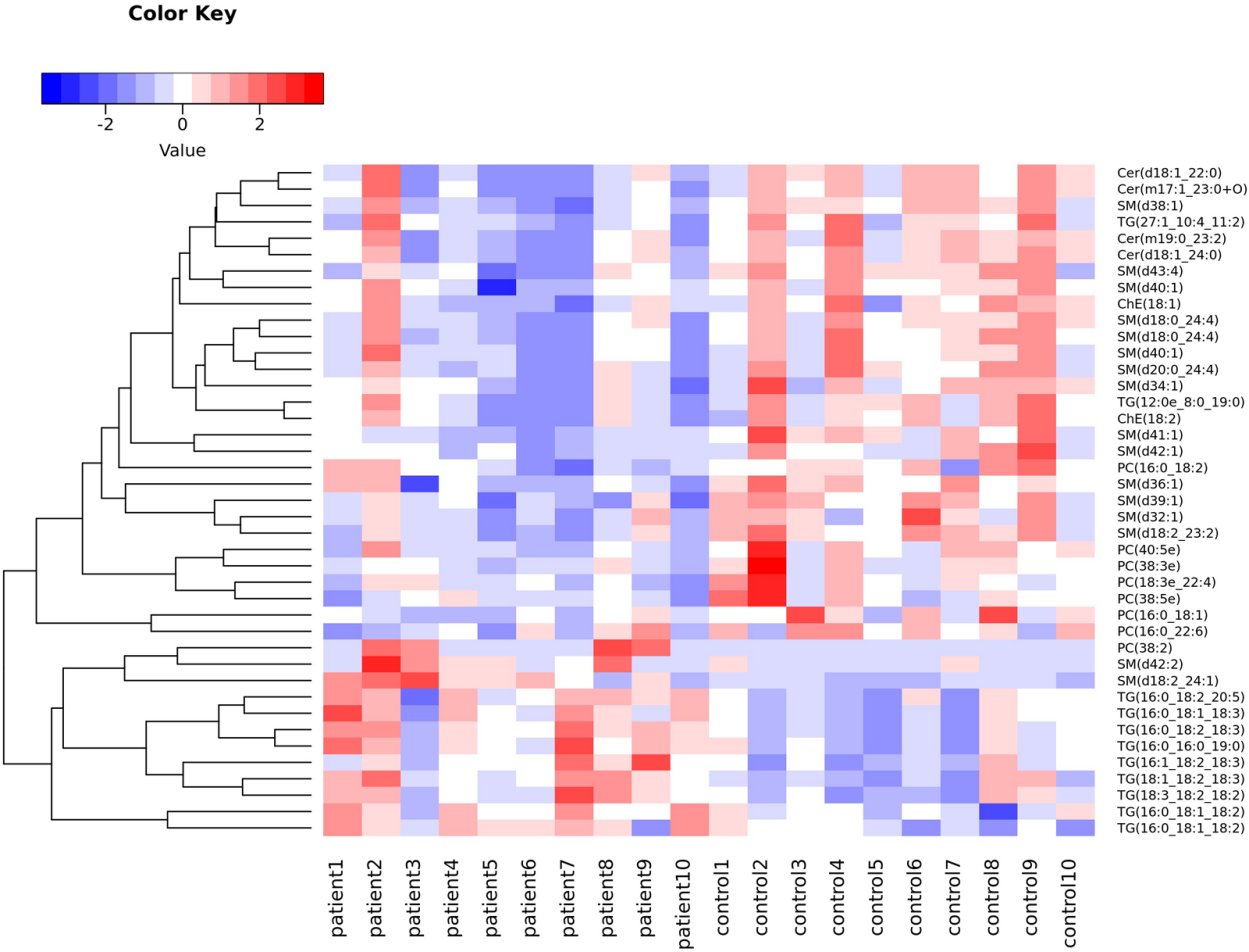
Heatmap of DELs.

**Figure 7:**
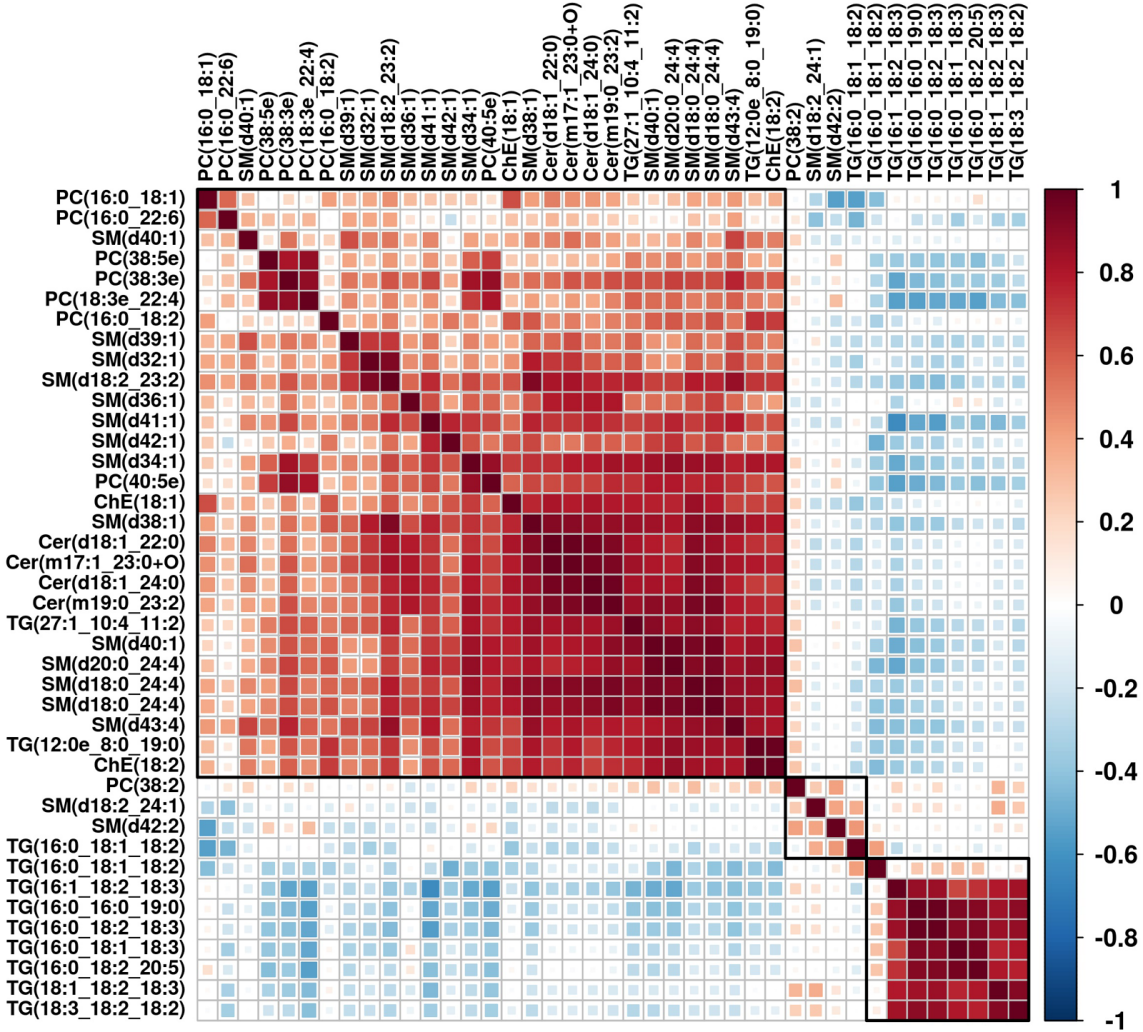
Correlation analysis of DELs.

A permutation test was also conducted and showed that the model had no overfitting. To identify differentially expressed lipids (DEL), the same criteria used to find DEM were applied. As a result, 41 DELs were identified, as detailed in Table 2. Clustering analysis showed a clear separation between CPP patients and controls and the heatmap is shown in Figure 6. Correlation analysis was conducted to evaluate the metabolic proximities between DELs. Positive correlation suggests that lipids share the same synthesis pathway and negative correlation suggests that the lipolysis of a lipid contributes to the synthesis of another lipid. The visualization of correlation analysis is presented in Figure 7. Chain length analysis and lipid saturation analysis yielded similar results, and significant differences were only observed for (O-acyl) ω-hydroxy fatty acids (OAHFA) between the two groups.

**Table 2:** List of differentially expressed lipids in CPP patients and controls.

Lipid	Class	Formula	Fold change	*P*-value	VIP
PC(16:0_18:1) + HCOO	PC	C43 H83 O10 N1 P1	0.812	0.031	5.996
PC(16:0_18:2) + HCOO	PC	C43 H81 O10 N1 P1	0.859	0.044	4.848
Cer(d18:1_22:0) + HCOO	Cer	C41 H80 O5 N1	0.749	0.011	1.015
SM(d32:1) + HCOO	SM	C38 H76 O8 N2 P1	0.653	0.012	1.118
SM(d34:1) + HCOO	SM	C40 H80 O8 N2 P1	0.863	0.019	3.583
SM(d36:1) + HCOO	SM	C42 H84 O8 N2 P1	0.805	0.016	1.780
Cer(d18:1_24:0) + HCOO	Cer	C43 H84 O5 N1	0.760	0.007	1.711
SM(d38:1) + HCOO	SM	C44 H88 O8 N2 P1	0.782	0.004	1.374
SM(d39:1) + HCOO	SM	C45 H90 O8 N2 P1	0.486	0.001	1.363
SM(d40:1) + HCOO	SM	C46 H92 O8 N2 P1	0.813	0.044	1.846
SM(d40:1) + HCOO	SM	C46 H92 O8 N2 P1	0.736	0.041	2.578
SM(d41:1) + HCOO	SM	C47 H94 O8 N2 P1	0.644	<0.001	2.092
SM(d42:1) + HCOO	SM	C48 H96 O8 N2 P1	0.716	0.029	2.012
Cer(m17:1_23:0 + O) + HCOO	Cer	C41 H80 O5 N1	0.733	0.008	1.094
SM(d18:2_23:2) + H	SM	C46 H88 O6 N2 P1	0.560	0.001	1.428
SM(d42:2) + H	SM	C47 H94 O6 N2 P1	4.630	0.046	11.407
SM(d18:2_24:1) + H	SM	C47 H92 O6 N2 P1	3.939	0.002	1.845
SM(d18:0_24:4) + H	SM	C47 H90 O6 N2 P1	0.841	0.021	2.350
SM(d18:0_24:4) + H	SM	C47 H90 O6 N2 P1	0.740	0.007	1.457
SM(d43:4) + H	SM	C48 H92 O6 N2 P1	0.671	0.002	1.972
SM(d20:0_24:4) + H	SM	C49 H94 O6 N2 P1	0.724	0.013	2.496
TG(12:0e_8:0_19:0) + H	TG	C42 H83 O5	0.873	0.034	3.308
ChE(18:1) + NH4	ChE	C45 H82 O2 N1	0.868	0.044	1.936
TG(27:1_10:4_11:2) + NH4	TG	C51 H88 O6 N1	0.827	0.029	1.600
ChE(18:2) + NH4	ChE	C45 H80 O2 N1	0.871	0.034	4.599
TG(16:0_16:0_19:0) + Na	TG	C54 H104 O6 Na1	1.734	0.015	2.846
TG(16:0_18:1_18:2) + NH4	TG	C55 H104 O6 N1	1.372	0.032	8.225
TG(16:0_18:1_18:2) + NH4	TG	C55 H104 O6 N1	1.772	0.034	10.762
TG(16:0_18:1_18:3) + NH4	TG	C55 H102 O6 N1	1.347	0.033	6.438
TG(16:0_18:2_18:3) + NH4	TG	C55 H100 O6 N1	1.786	0.005	5.088
TG(16:1_18:2_18:3) + NH4	TG	C55 H98 O6 N1	1.770	0.033	1.682
TG(18:1_18:2_18:3) + NH4	TG	C57 H102 O6 N1	1.810	0.016	4.711
TG(18:3_18:2_18:2) + NH4	TG	C57 H100 O6 N1	1.642	0.029	2.053
TG(16:0_18:2_20:5) + H	TG	C57 H97 O6	1.198	0.035	1.229
PC(38:2) + H	PC	C46 H89 O8 N1 P1	4.398	0.034	3.058
PC(38:3e) + H	PC	C46 H89 O7 N1 P1	0.617	0.043	1.480
PC(38:5e) + H	PC	C46 H85 O7 N1 P1	0.734	0.036	1.162
PC(16:0_22:6) + H	PC	C46 H81 O8 N1 P1	0.873	0.046	2.120
PC(40:5e) + H	PC	C48 H89 O7 N1 P1	0.744	0.028	1.265
PC(18:3e_22:4) + H	PC	C48 H85 O7 N1 P1	0.740	0.026	1.275
Cer(m19:0_23:2) + H	Cer	C42 H82 O2 N1	0.767	0.009	1.284

## Discussion

The present study was a comprehensive analysis of untargeted metabolomics and lipidomics in plasma samples collected from CPP patients and controls. The analysis revealed that several metabolites and lipids were differentially expressed between the two groups. The findings may aid understanding of the mechanism of CPP and indicate biomarkers for diagnosis.

The most striking finding was the 5.251-fold elevation of ciprofloxacin in CPP patients compared with controls. Ciprofloxacin is a fluoroquinolone antibiotic used to treat infections caused by bacteria such as pneumonia, typhoid fever, and infectious diarrhea. Fluoroquinolone antibiotics are capable of disrupting the catalytic mechanism of topoisomerase IV and DNA gyrase, and lead to single-stranded and double-stranded DNA breaks (20). Side effects of fluoroquinolone are common and involve several biological systems. Therefore, the application of fluoroquinolone is prohibited in certain populations, especially pregnant women.

An association between fluoroquinolone and CPP has been reported in a cross-sectional study in China. In this study, the detection rate of fluoroquinolone in children with CPP was significantly higher than that in children without CPP (21). Although antibiotics are prescription-only medicines in developed countries including the United States and Canada, they can be easily purchased from pharmacies in China without a prescription. In addition, parents in China often use antibiotics to treat infections in their children because they believe antibiotics are very effective. These two factors have contributed to the overuse of antibiotics in China, and this overuse may be one explanation for the increase in the incidence of CPP.

We also observed a significant elevation of pregnenolone sulfate in CPP patients and the FC reached 2.397. Pregnenolone sulfate is a steroid metabolite with a plethora of functions. Pregnenolone is synthesized from cholesterol by a mitochondrially localized cytochrome P450 side-chain cleavage enzyme and then some of the pregnenolone is converted to pregnenolone sulfate by sulfonation of cytosolic sulfotransferase enzymes (22). Pregnenolone sulfate plays a critical role in the synthesis of steroid hormones, including estradiol and progesterone. It also modulates potassium channels, nicotinic acetylcholine receptors, and voltage-gated sodium channels (23). The elevation of pregnenolone sulfate in the current study may indicate upregulated synthesis of steroid hormones, which is consistent with the definition of CPP. Therefore, it could serve as a biomarker during screening for CPP.

Interestingly, the results showed that levels of propyl pyrazole triol, an estrogen receptor alpha agonist, were approximately four time higher in CPP patients than controls. In an animal model, the application of propyl pyrazole triol was very effective in stimulating uterine weight gain and upregulating complement 3 gene expression, as well as stimulating other physiologically relevant estrogen-induced tissue responses (24). Similar to the elevation of pregnenolone sulfate, an increase of propyl pyrazole triol contributes to pubertal changes, such as uterine growth.

KEGG pathway analysis showed that DEMs were enriched in several pathways, including steroid hormone biosynthesis (which is explained by the above-mentioned metabolites) and histidine metabolism. The key DEM in histidine metabolism we identified was 3-methylhistamine, which was increased three-fold in CPP patients. This histamine metabolite is an index of the rate of muscle protein breakdown. Consequently, its measurement in urine is often used to evaluate the physical activity of professional athletes. It originates from the consumption of protein-rich foods, for example, meat and soy products. A previous study revealed that increased excretion of 3-methylhistamine was associated with food consumption (25). In the contemporary livestock industry, hormones such as estrogens are often administered to growing cattle to promote growth, enabling them to be ready for production earlier. As a result, the residue of hormones in meat products has become a serious problem in food safety. Xu et al. (26) conducted an investigation into the estrogen residue of livestock manure and found that estrogen levels were highest in the manure of finishing pigs, followed by growing pigs, and piglets. Our findings may reflect high levels of estrogen residues in meat products, promoting CPP in susceptible individuals with high meat consumption.

We also performed lipidomics to investigate changes in lipids in CPP patients. In total, 41 DELs were identified and according to the results of Student's t-tests, most of PC, SM and ChE were decreased in CPP patients while most TGs were upregulated. TG elevation suggests that CPP patients may be overweight or consume a diet high in meat products, which are also contaminated with antibiotics and estrogen residues. In the correlation analysis, lipids within the same class showed strong correlation, suggesting that they interact with each other. The function of some DELs should be further explored, especially those in SM, which have an important function in neuronal activity.

## Conclusion

In conclusion, we conducted untargeted metabolomics and lipidomics analyses in plasma samples collected from CPP patients and controls. The data analysis revealed several DEMs and DELs that hold potential to be biomarkers. These DEMs and DELs could also improve understanding of the underlying mechanisms of CPP. Several KEGG pathways were enriched in DEMs and could provide valuable information concerning CPP. The increasing incidence of CPP may be associated with the overuse of antibiotics and the consumption of meat products that contain high level of estrogen. Moreover, obesity and fat accumulation caused by increased food consumption also contributes to the development of CPP. The major limitation of our study is the sample size, considering the incidence, the number of children, and potential hospital visit, as well as the multi-omics approach we applied, we are able to conduct such study in a large sample size. Moreover, the sample size limits the possibility of introducing factors such as social-economic status, dietary intake to our analysis. The findings should be validated in a larger sample size. In addition, cell assays are required to clarify the functional roles of the DEMs and DELs in the development of CPP.

## Data Availability

The raw data supporting the conclusions of this article will be made available by the authors, without undue reservation.
